# Separation of Macro- and Micro-Texture to Characterize Skid Resistance of Asphalt Pavement

**DOI:** 10.3390/ma17204961

**Published:** 2024-10-11

**Authors:** Tao Xie, Enhui Yang, Qiang Chen, Junying Rao, Haopeng Zhang, Yanjun Qiu

**Affiliations:** 1 College of Civil Engineering, Guizhou University, Guiyang 550025, China; xiet@gzu.edu.cn; 2 Guizhou University Survey and Design Institute Co., Ltd., Guizhou University, Guiyang 550025, China; 3 School of Civil Engineering, Southwest Jiaotong University, Chengdu 610031, China; ehyang@swjtu.edu.cn (E.Y.); qchen@swjtu.edu.cn (Q.C.); yjqiu@swjtu.edu.cn (Y.Q.); 4 Highway Engineering Key Laboratory of Sichuan Province, Southwest Jiaotong University, Chengdu 610031, China; 5 Research Center of Space Structure, Guizhou University, Guiyang 550025, China; jyrao@gzu.edu.cn

**Keywords:** asphalt pavement, texture separation, macro-texture characteristics, micro-texture features, Monte Carlo algorithm, skid resistance

## Abstract

The skid resistance of asphalt pavement is an important factor affecting road safety. However, few studies have characterized the contribution of the macro- and micro-texture to the skid resistance of asphalt pavement. In this paper, the generalized extreme studentized deviate (GESD) and neighboring-region interpolation algorithm (NRIA) were used to identify and replace outliers, and median filters were used to suppress noise in texture data to reconstruct textures. On this basis, the separation of the macro- and micro-texture and the Monte Carlo algorithm were used to characterize the skid resistance of asphalt pavement. The results show that the GESD method can accurately identify outliers in the texture, and the median filtering can eliminate burrs in texture data while retaining more original detail information. The contribution of the macro-texture on the skid resistance is mainly attributed to the frictional resistance caused by the adhesion and elastic hysteresis, and the main contribution of the micro-texture is a micro-bulge cutting part in the friction mechanism. This investigation can provide inspiration for the interior mechanism and the specific relationship between the pavement textures and the skid resistance of asphalt pavement.

## 1. Introduction

The friction between the tire and the pavement surface can lead to sliding resistance and energy loss, and it depends on the contact state between the tire and the pavement surface, which starts from the top of a few rough micro-bulges on the two surfaces [1,2]. The contact pressure can cause plastic deformation at the contact point [3,4,5], and the contact points between the tire and the road surface are not continuous, but discrete. The contact state determines the friction between the tire and the pavement, which mainly includes intermolecular, adhesion, elastic hysteresis, and micro-bulge cutting interactions [6]. In addition, some types of aggregate could be too abrasive and create ‘too good adhesion’, which can contribute to accelerated tire wear and the formation of micro-rubber particles [7]. Therefore, the skid resistance of the pavement is affected by several factors such as pavement texture, material properties, vehicle operating status, tire pressure, and road pollutants [8].

Although there are many factors needed to be considered, it is generally believed that the pavement textures play a key role in the skid resistance of asphalt pavement [9,10,11]. On the one hand, the textures affect the actual contact area between the tire and the pavement surface and have an important influence on the intermolecular force and adhesion in friction [10]. On the other hand, the texture directly participates in the elastic hysteresis and cutting process of micro-bulges, which influences the size of these two components in the friction [9,11]. Therefore, it is very important to accurately collect and comprehensively evaluate the pavement texture topography for the investigation of friction characteristics between the tire and the pavement surface.

The deviation between the surface and the horizontal plane of the pavement is defined as the pavement texture, and the wavelength is the minimum interval of a periodically repeated part, which can determine different scale ranges and pavement textures [12,13]. The Permanent International Association of Road Congress (PIARC) divides pavement texture into four categories according to the wavelength range, including unevenness, macrostructure, macro-texture, and micro-texture, where the micro-texture is less than 0.5 mm, the macro-texture is in the range of 0.5–50.0 mm, the macrostructure is between 50 and 500 mm, and the uneven texture is more than 500 mm [14,15]. On this basis, some researchers try to measure the macro-texture parameters of asphalt pavement and establish a prediction model of the skid resistance for asphalt pavement. Then, the parameters of macro-texture can be termed as a direct input parameter of the prediction model [16,17]. Although these models can make full use of laboratory data to avoid some troubles caused by field measurements to predict the skid resistance of asphalt pavement at a certain extent, the correlation between the results of the prediction model and field measured data is not good according to the standard of correlation coefficients [18,19,20].

Based on the above literature, scholars mainly obtain macro-texture topography through various methods to evaluate the skid resistance of asphalt pavement. However, there are few studies on comprehensively characterizing the skid resistance of asphalt pavement through the separation of the macro- and micro-texture. In this paper, three-dimensional laser image technology was used to extract and separate high-precision feature information of the macro- and micro-texture for asphalt pavement, and the Monte Carlo expectation method was performed to calculate the average structure depth. On this basis, the prediction relationship between pavement texture features and the skid resistance is also established. This study can provide some inspirations for predicting the skid resistance of asphalt pavement and the corresponding mechanism.

## 2. Research Objectives

**Figure 1:**
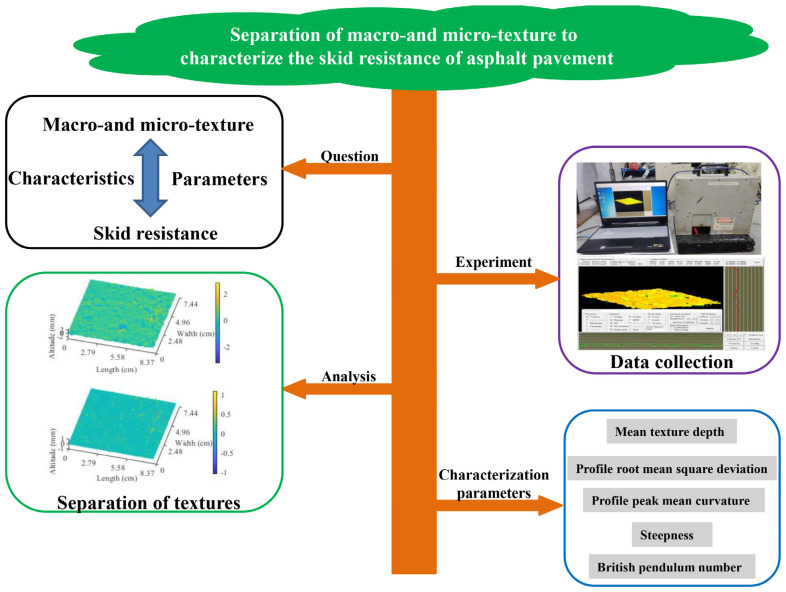
Diagram of research objectives and methods.

This paper is to comprehensively investigate contributions of macro- and micro-texture to the skid resistance of asphalt pavement through the separation of macro- and micro-texture and the Monte Carlo algorithm, which can reveal the interior mechanism and the specific relationship between pavement textures and the skid resistance of asphalt pavement. The diagram of research objectives and the method are shown in Figure 1, and there are four objectives in this paper as follows.

(1) To identify and replace the outliers using the generalized extreme studentized deviate (GESD) and neighboring-region interpolation algorithm (NRIA).
(2) To suppress noise in the pavement textures data and reconstruct the road texture through the median filter.
(3) To separate the macro- and micro-texture in asphalt pavement according to their corresponding frequency.
(4) To establish the relationship between the characteristics of the macro- and micro-texture and the skid resistance.

## 3. Materials and Methods

### 3.1. Raw Materials

The origin bitumen used in this paper originated from the Mianyang Bitumen Company, and the basic physical properties of the origin bitumen are shown in Table 1. In addition, the styrene–butadiene–styrene (SBS) was also purchased from the Mianyang Bitumen Company. In order to investigate the changes of the macro- and micro-texture characteristics and the skid resistance of the asphalt pavement, three typical-graded asphalt mixtures including dense-graded asphalt concrete (AC), stone mastic asphalt (SMA), and open-graded friction course (OGFC) were selected, and the rutting slabs with different maximum nominal particle sizes were made for the experiment. The maximum nominal particle size of AC and SMA was 16 mm, 13 mm, and 10 mm, and the maximum nominal particle size of OGFC was 13 mm and 10 mm. The gradation design of the mixture is shown in Table 2. The size of the rutting plate specimen was 300 mm × 300 mm × 50 mm, and the lignocellulose was doped into the asphalt mixture, and the added content was 0.3% of weight of the asphalt mixture. 

### 3.2. Data Collection and Processing

**Figure 2:**
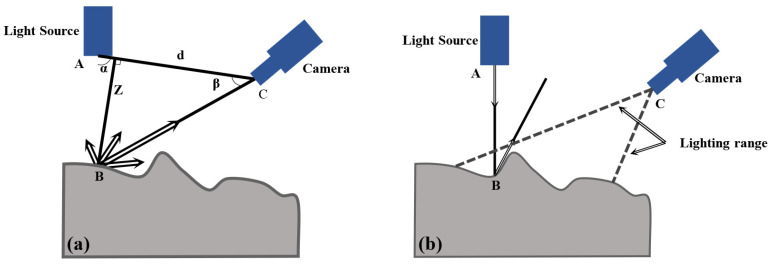
The principle of laser triangulation for the LS-40 equipment (**a**) Laser beam came from a known angle of the instrument, (**b**) Measurement points exceeding the measurement range of the instrument.

The 3D laser scanning equipment (LS-40), Artec 3D, Senningerberg, Luxembourg, used in the study relied on a built-in laser transmitter to emit laser light, and moved the laser beam on the surface of the object [27,28]. The reflected laser beam information was received by the high-resolution camera, and the principle of laser triangulation was used to analyze the laser beam information, which is shown in Figure 2. As shown in Figure 2a, the laser beam generated by the laser transmitter came from a known angle of the instrument, and hit the surface of the object at point B. After one reflection, the reflection intensity depended on the type of surface of the object, and part of the reflected light was received by camera C. There was a phenomenon that it could not accurately measure the surface elevation information of the object [29]. On the one hand, this phenomenon was caused by part of the reflected light not being received by the camera. On the other hand, it was attributed to the elevation of measurement points exceeding the measurement range of the instrument (Figure 2b). The results of the final elevation would be displayed as values much larger than the standard range. Therefore, it was necessary to use NRIA to replace the erroneous results in the original data.

Therefore, the elevation data of the three-dimensional discrete point cloud of point B can be calculated as follows [27]:

(1) $$Z=\frac{d}{\cot\alpha +\cot\beta }$$

where *d* is the distance between two points A and C, *α* is angle between the incident ray and the AC line, and *β* is angle between the reflected ray and the AC line. Each experiment was repeated three times and the average value was taken as the experimental value.

**Figure 3:**
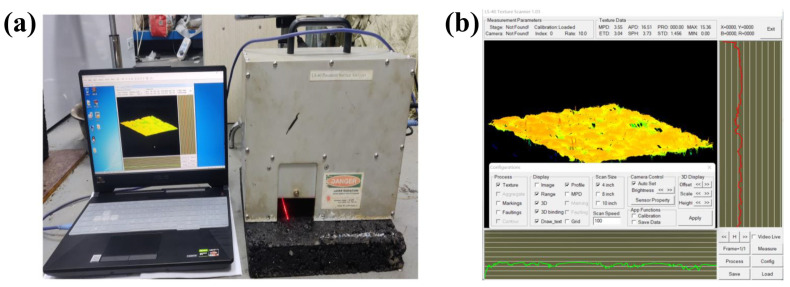
Surface texture elevation data collected by the LS-40 equipment of (**a**) field collection and (**b**) data collection.

The LS-40 equipment collected the linear profile from the target surface. Each measurement scanned a total of 2048 profiles, and each profile line contained 2048 data points, which covered an area of 10.16 cm × 11.43 cm with a horizontal accuracy of 0.05 mm. The LS-40 equipment could measure the elevation texture information within 12.5 mm of the top and bottom of the surface because the instrument was placed on a surface of the specimen with a vertical accuracy of 0.01 mm [28]. The scanning results were displayed in the form of elevation data, which is illustrated in Figure 3.

**Figure 4:**
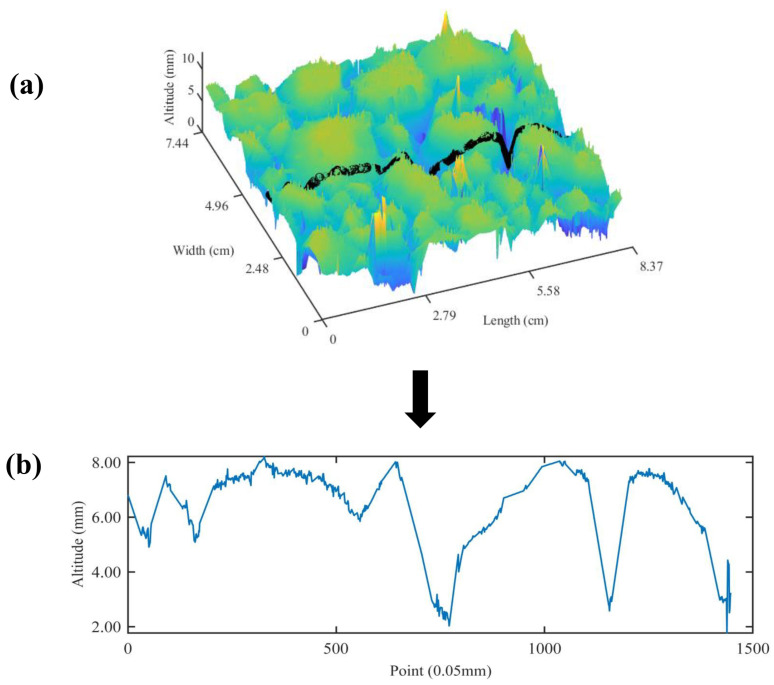
Schematic diagram: (**a**) color map; (**b**) waveform of original texture.

**Figure 5:**
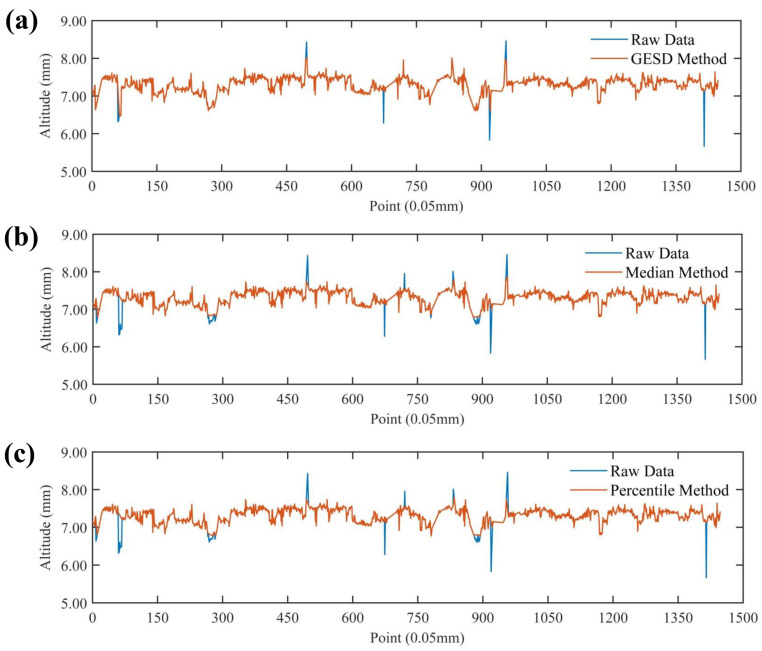
Comparison of outlier identification methods: (**a**) GESD method; (**b**) median method; (**c**) parametric method.

The scanned contour of the LS-40 equipment was cut into 1448 × 1448 data points, and the original texture data of the contour scanned by the LS-40 equipment is shown in Figure 4. It can be seen from Figure 5 that there are outliers and signal noise in the original texture data, and there are some outliers and glitches in the 3D and 2D images.

#### 3.2.1. Outlier Processing

Compared with most of the normal data, there were significant differences in the outliers [30]. The elevation values of the pavement textures usually changed continuously, but the measured elevation value deviated from larger values due to the reflective, oily, and occlusion characteristics of the specimen surface. The existence of outliers will lead to the inexact reconstruction and evaluation of topographical characteristics for pavement textures. Therefore, it is necessary to identify and replace the outliers. 

Figure 5 demonstrates the comparative effect of each outlier identification method, and it can be seen that the median and parametric methods identify part of the pavement textures contour information as outliers in the process of identifying the pavement texture outliers, while the GESD method can accurately identify the outliers of pavement textures and the elevation profile information of pavement textures is relatively completely preserved compared with the median and parametric methods. Therefore, the outlier recognition effect is better.

Based on this, the outlier identification is performed on the raw texture data in the experiment using the GESD method, and the identified outliers are replaced by the NRIA of the adjacent non-outliers as shown in Equation (2) [31]

(2) $$z_{i}=z_{n}+\frac{z_{n}-z_{m}}{n-m}(i-m)$$

where *i* is the identified outlier number, *n* is the data number of the non-outlier immediately before *i*, *m* is the data number of the non-outlier immediately after *i*, *z_i_* is the replacement value of the outlier at *i*, *z*_n_ is the contour elevation value of number *n*, and *z*_m_ is the contour elevation value of number *m*. 

#### 3.2.2. Filter Denoising

The noise is the interference superimposed on the real value due to a system error and signal transmission, and the actual measured pavement texture data often contains real and noise data at the same time, which also causes the collected original road surface texture to appear frizzy and oscillating [32]. The filtering can remove the noise signal in the original texture information, and both the mean and median filtering belong to the smoothing methods in the spatial domain [33]. The mean filtering is a type of linear filtering, which replaces element values in the center of the template by calculating the average value of elements within the coverage of the template to achieve the purpose of denoising. Therefore, the filtering effect of the mean filter is related to the size of the selected template radius. It was reported that the mean filtering had advantages of a fast running speed, a simple algorithm, and easy implementation, and the calculation formula of the mean filter is as follows [33]:

(3) $$g\left(x,y\right)=\frac{1}{{\left(2i+1\right)}^{2}}\sum_{i=-r}^{r}\sum_{j=-r}^{r}I\left(x+i,y+i\right)$$

where *g* (*x*, *y*) is the filtered element value, *I*(*x*, *y*) is the original signal element value, and *i* is the calculation window template radius. When *i* = 1, the window template is as follows:
1/91/91/91/91/91/91/91/91/9

As a nonlinear filter, the median filter sorts the element values in a window template, and replaces the element values in the center of the template with a median value of the elements in the window template (Equation (4)), which shows good noise smoothing properties [33]

(4) $$g(x,y)=med\left\{f\left(x-k,y-l\right)\right\}$$

where *f*(*x*, *y*) is the original signal element value, and *k* and *l* are both calculation window template sizes. 

The wavelet threshold denoising is performed by setting a threshold in each wavelet decomposition scale. The wavelet coefficient part is judged as a noise signal because the decomposed wavelet coefficient is less than the threshold value. Then, the original signal is reconstructed by a new wavelet coefficient to achieve the purpose of denoising [34]. 

**Figure 6:**
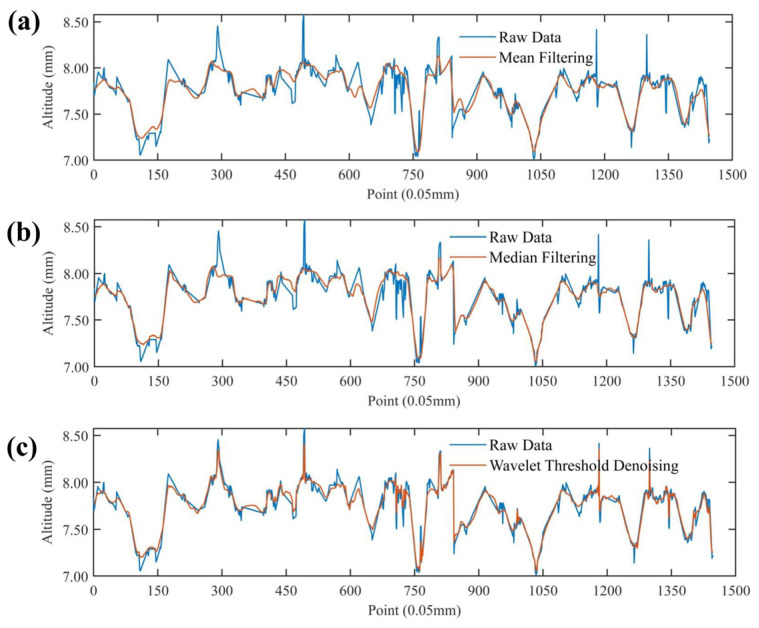
Comparison of the filter denoising methods: (**a**) mean filtering; (**b**) median filtering; (**c**) wavelet threshold denoising.

**Figure 7:**
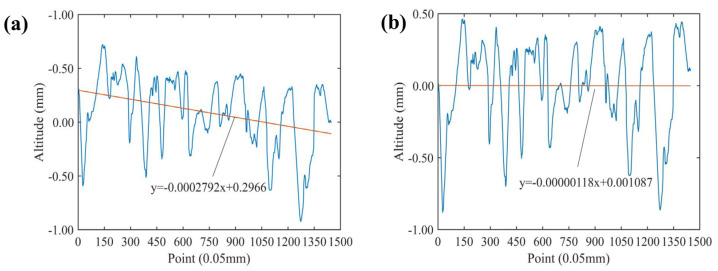
The pavement profile (**a**) before and (**b**) after tilt correction.

Figure 6 compares the filtering effects of mean and median filtering and wavelet threshold denoising. As shown in Figure 7, the wavelet threshold denoising is the closest to the original signal, and retains most of the characteristics of the original signal, which also leads to incomplete filtering of the wavelet threshold denoising such as some glitches. Both mean and median filtering can eliminate the glitch points, and the median filter is in the middle of the wavelet threshold denoising and mean filtering to the extent that it is close to the original signal. The mean filtering is calculated by the average value of all elements in the window. This will discard signal details in the filtering process, and the image is blurred in a larger range. Therefore, the median filtering retains more original details than the mean filtering.

#### 3.2.3. Tilt Correction

In addition, the placement of the mixture specimen cannot reach a completely horizontal state, which will cause the reconstructed surface to appear inclined and also affect the calculation of the pavement texture index and the evaluation of the texture topography. Therefore, it is necessary to eliminate the slope and offset errors of the elevation profile data, and the specific implementation formula of the tilt correction is as Equations (5)–(7) [34]

(5) $$H_{i}=h_{i}-b_{1}i-b_{0}\;i=0,\ldots ,n-1$$

(6) $$b_{1}=\frac{12\sum_{i=0}^{n-1}ih_{i}-6(n-1)\sum_{i=0}^{n-1}h_{i}}{n(n+1)\left(n-1\right)}$$

(7) $$b_{0}=\frac{1}{n}\sum_{i=0}^{n-1}h_{i}-b_{1}\cdot \frac{n-1}{2}$$

where *i* is the number of the contour elevation data point; *H_i_* is the slope correction contour elevation corresponding to number *i*; *h_i_* is the height of the original contour corresponding to number *i*; *N* is the total number of collection points for the contour line; *b*_1_ is the elimination coefficient of slope error, which can be calculated as Equation (6); and *b*_0_ is the bias error item calculated by Equation (7). The pavement profile before and after the eliminated slope error and offset error are demonstrated in Figure 7.

**Figure 8:**
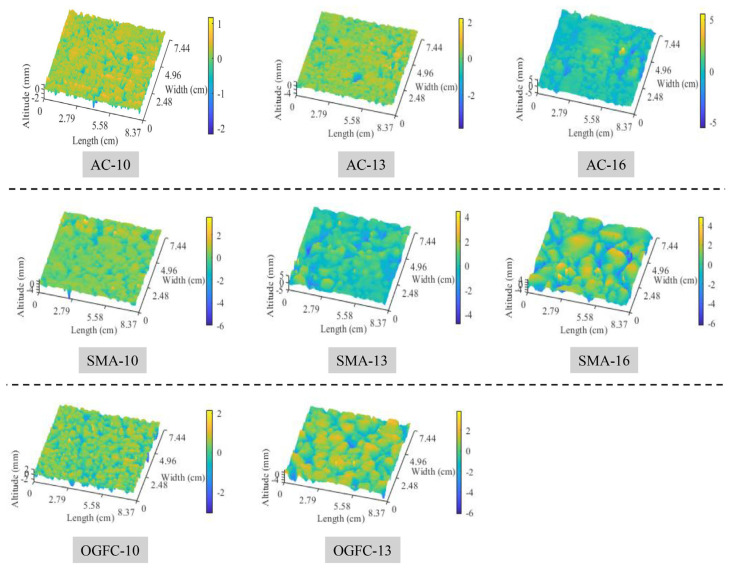
3D reconstructed surfaces of various types of specimens.

The asphalt pavement surface texture is reconstructed after intercepting the collected original texture data, identifying and replacing outliers, filtering to remove noise signals, and inclination correction [35]. Figure 8 illustrates the three-dimensional reconstructed surfaces for various types of specimens, and it can be seen that the height distribution becomes uneven as the types of specimen changes from the AC to OGFC specimen, and the OGFC-13 specimen presents the largest elevation difference in the same reconstructed surface.

### 3.3. Separation of Macro- and Micro-Texture

#### 3.3.1. Separation Details

The surface texture of asphalt pavement always contains the texture of various wavelengths [36], and the macro- and micro-texture of pavement have different contributions on the friction mechanism between the tire and the pavement surface. Therefore, to investigate the influence of macro- and micro-texture on the skid resistance of pavement, it is necessary to separate and extract the macro- and micro-texture from the pavement surface textures according to wavelength differences. As a basic operation in signal processing, Fourier transform can realize the conversion from the space to the frequency domain, and it is widely used in signal processing, optics, probability, statistics, and other fields. Therefore, the Fourier-transform method was used to process the texture data of the road surface to convert the texture data from the spatial domain to the frequency domain in this paper, and the Fourier transform is calculated as follows [36]:

(8) $$F(u,v)=\iint f(x,y)e^{-j2\pi (ux+vy)}dxdy$$

**Figure 9:**
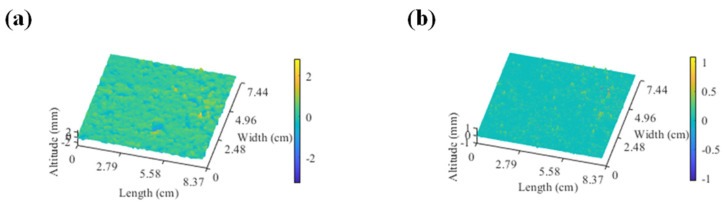
3D reconstruction of (**a**) macro-texture and (**b**) micro-texture obtained from AC-13.

In this paper, a sliding window-based finite impulse response (FIR) digital filter was designed to separate and extract the macro- and micro-texture from collected pavement texture data and the design of the FIR digital filter selects the Hamming window. The band-pass filter was used to extract the macroscopic texture from the road surface, and the upper and lower limit frequencies of the band-pass filter are 2 mm^−1^ and 0.2 mm^−1^, respectively. The high-pass filter is designed to extract the microscopic texture and the corresponding cut-off frequency and filter order of the high-pass filter is 0.02 mm^−1^ and 400, respectively. Based on this, the results of the macro- and micro-texture separated from the 3D reconstructed pavement of AC-13 are demonstrated in Figure 9.

**Figure 10:**
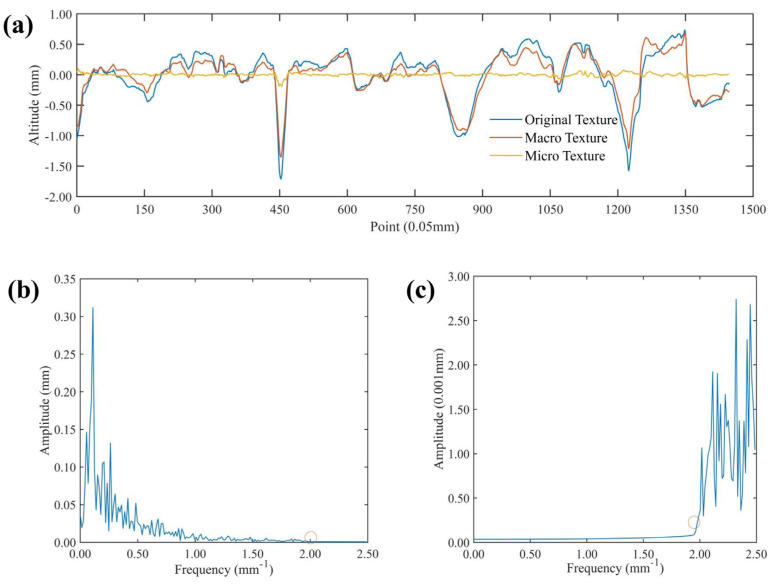
Pavement macro- and micro-texture separation and frequency domain map of (**a**) the contour line, (**b**) the frequency domain of the macro-texture, and (**c**) the frequency domain of the micro-texture from AC-13.

Figure 10 takes one profile line as an example to illustrate the separation results of the pavement macro- and micro-texture from AC-13. According to Figure 10a, it can be seen the macro-texture of the pavement is basically consistent with the profile shape of the overall profile line, and the macro-texture is smaller than that of the pavement outline. Figure 10b,c show the frequency information of the macro- and micro-texture after frequency domain filtering, respectively. The amplitude of the part with a frequency greater than 2 mm^−1^ is 0 in the frequency domain map of the macroscopic texture, while the amplitude of the part with a frequency less than 2 mm^−1^ is also close to 0 for the frequency domain map of the microscopic texture. The above results show that the pavement texture is converted from the spatial domain to the frequency domain using the fast Fourier transform, and the filter is designed according to the frequency domain information corresponding to the wavelength of the macro- and micro-texture, which can effectively separate the macro- and micro-texture from the pavement textures.

#### 3.3.2. Pavement Texture Indexes

The texture characteristics of asphalt pavement have a great influence on the contact behavior between the tire and the pavement surface. Therefore, it is necessary to comprehensively evaluate pavement texture characteristics from different angles and scales. On the basis of existing research [37,38], a number of parameters are selected from aspects of the pavement topography height, protruding body shape, comprehensive distribution, and fractal characteristics to comprehensively evaluate the macro- and micro-texture of pavement. This can also analyze the correlation between texture parameters and swing value, which aims to investigate the texture characteristics of asphalt pavement and its influence on the skid resistance of asphalt pavement.

The root-mean-square deviation (RMSD) *R_q_* is a parameter describing texture height properties, which characterizes the discreteness of the pavement profile and weights amplitude of the larger absolute value in data. This will further highlight the influence of the larger absolute amplitude on the texture height feature, and the calculation of *R_q_* of the discrete texture data is as follows [37]:

(9) $$R_{q}=\sqrt{\frac{1}{N}\sum_{i=1}^{n}{z_{i}}^{2}}$$

The Monte Carlo expectation method is used to process the macro- and micro-texture data of pavement to obtain the mean texture depth (MTD). This method is used to calculate statistical eigenvalues as numerical solutions of practical problems through continuous sampling and gradual approximation based on the theory of probability and statistics, which has the advantage of a clear program structure and the convergence speed is not affected by the dimension of the problem [39,40]. The process of calculating pi is as follows. The radius of the circle is unit 1, and points are randomly cast into the circumscribed square area of the circle. The total number of cast points is *m*, and the number of cast points recorded in the circle is *a*, then the approximate value of pi can be obtained as follows [39]:

(10) $$\pi =\frac{4a}{m}$$

**Figure 11:**
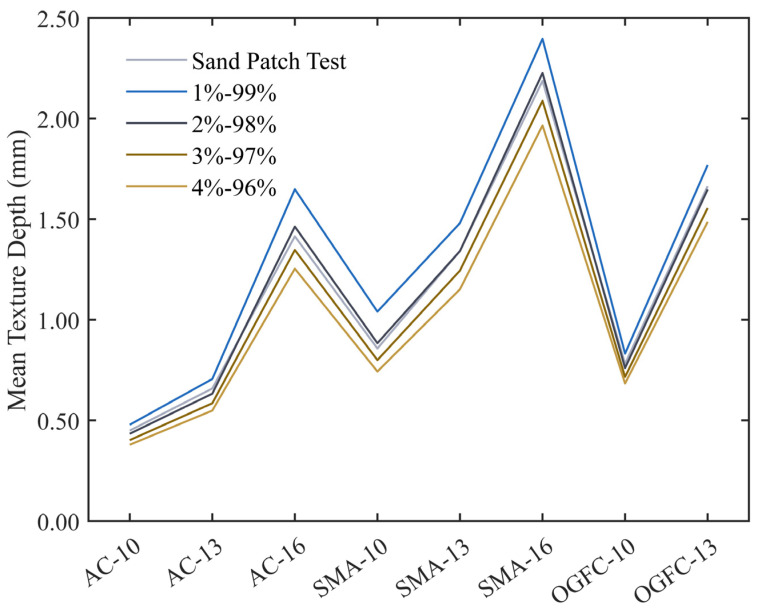
The MTD calculated by the sand patch and the Monte Carlo expectation methods.

**Figure 12:**
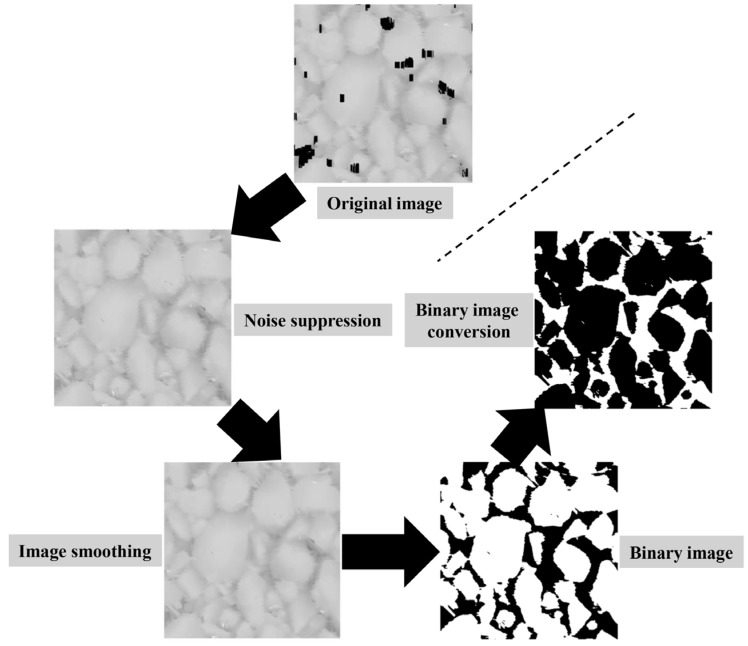
Schematic diagram of image segmentation of the specimen.

During this process, the parametric points of the overall data are considered as the upper and lower limits of the rectangular frame. It was reported that the sand patch method could also be used to exactly calculate MTD, but the experimental process is not easy to operate manually compared with the Monte Carlo expectation method, and the calculation process could be found in previous literature [41,42]. Therefore, Figure 11 compares the MTD obtained by the sand patch method and the Monte Carlo expectation method with different parametric points. From Figure 12, the MTD calculated by the Monte Carlo expectation method is the closest to that obtained by the sand-laying method because the 98% and 2% parametric points are used as the upper and lower boundaries of the rectangular box, and more original elevation data information are retained at the same time. Therefore, the 98% and 2% parametric points of the overall data are used as the upper and lower limits of the Monte Carlo expectation method in this paper.

The peak half angle (PHA) *α* can characterize the particle shape of the protruding peak on the surface of the specimen, and the larger PHA indicates a wider and flatter protruding peak, which can be calculated as follows [42]:

(11) $$\alpha =\frac{1}{2}\left[\arctan\left|\frac{x_{i}-x_{i-1}}{y_{i}-y_{i-1}}\right|+\arctan\left|\frac{x_{i+1}-x_{i}}{y_{i+1}-y_{i}}\right|\right]i=2,3,\ldots ,n$$

where *x_i_* is the abscissa of the *i*-th extreme point, *x_i_*_+1_ and *x_i_*_−1_ are the abscissa corresponding to the bottom of the valley, and *y_i_* is the *i*-th extreme point elevation value as the *i*-th extreme point is the peak.

The mean peak curvature (MPC) *K* can reflect the sharpness of the profile peak of the micro-bulge, and the sharpness of micro-texture affects the cutting effect of the micro-bulge. The smaller *K* value indicates that the point where the road surface contacts rubber is more rounded, and the point where the road surface contacts the rubber is sharper because the *K* value is large. The LS-40 equipment collects the discrete elevation data points, and Equation (12) for the MPC of discrete vertices is as follows [43]:

(12) $$K_{i}=\left|\frac{\arctan\left(\frac{y_{i+2}-y_{i+1}}{x_{i+2}-x_{i+1}}\right)-\arctan\left(\frac{y_{i+1}-y_{i}}{x_{i+1}-x_{i}}\right)}{\sqrt{{\left(x_{i+1}-x_{i}\right)}^{2}+{\left(y_{i+1}-y_{i}\right)}^{2}}}\right|$$

The kurtosis parameter *R*_ku_ is used to evaluate the steepness of the texture curve, and mainly reflects characteristics of overall distribution for contour elevation data, which is determined as [43]

(13) $$R_{\mathrm{ku}}=\frac{1}{{R_{q}}^{4}}\cdot \frac{1}{M\times N}\sum_{i=1}^{N}\sum_{i=1}^{M}z^{4}(x_{i},y_{i})$$

where *M* and *N* are the number of lateral and longitudinal contours of the pavement, *R*_q_ is the RMSD of the contour, and *z* (*x*, *y*) is the elevation of the pavement contour.

The height change of the surface profile for the pavement is an approximate random process, which characterizes the self-similarity in a certain level [44]. The fractal analysis is a powerful tool for the investigation of irregular shaped objects and can be used to describe the fractal characteristics of the pavement texture. In this paper, the box counting method is used to calculate the fractal dimension of the pavement surface texture images. Firstly, the grayscale image scanned by the LS-40 equipment is smoothed through the median filter. Then, the segmentation threshold is usually selected using the Otsu method [45]. Finally, the image can be divided into the background and target in the process of using this threshold to convert the grayscale image into a binary image. The image segmentation process is shown in Figure 12.

According to the above method, the MTD, RMSD, PHA, MPC, and kurtosis of the profile peak are calculated for the macro- and micro-texture, respectively. Then, the fractal dimension of the road surface is calculated by using the box counting method according to the analysis of the pavement surface image.

## 4. Results and Discussion

### 4.1. Macroscopic Texture Features and Skid Resistance Analysis

#### 4.1.1. Height Characteristic Parameters

The macro-texture of asphalt pavement is related to the gradation type of the mixture and the maximum nominal particle size. Therefore, it is very important to discuss the influence of composition of the asphalt mixture on the macro-texture parameters of asphalt pavement and to analyze the relationship between the macro-texture parameters and the British pendulum number (BPN) of asphalt pavement.

**Figure 13:**
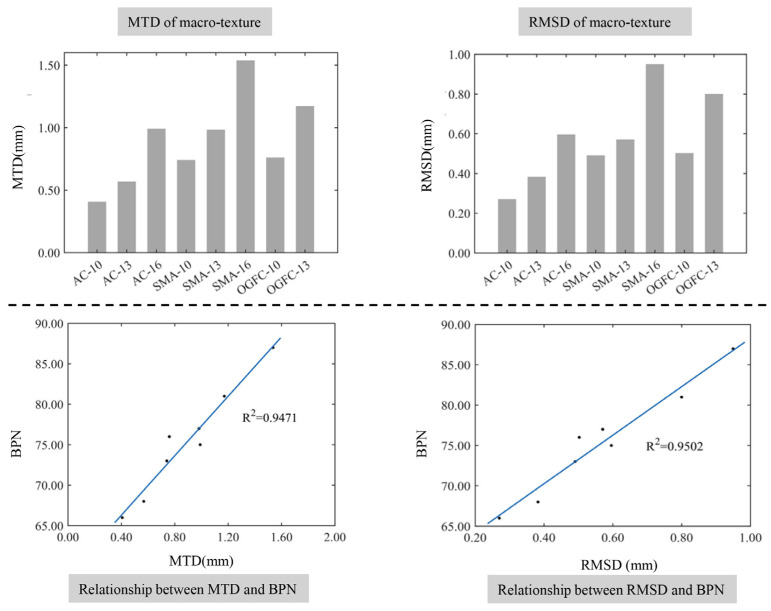
The relationship between height characteristics of the macro-texture and the BPN.

**Figure 14:**
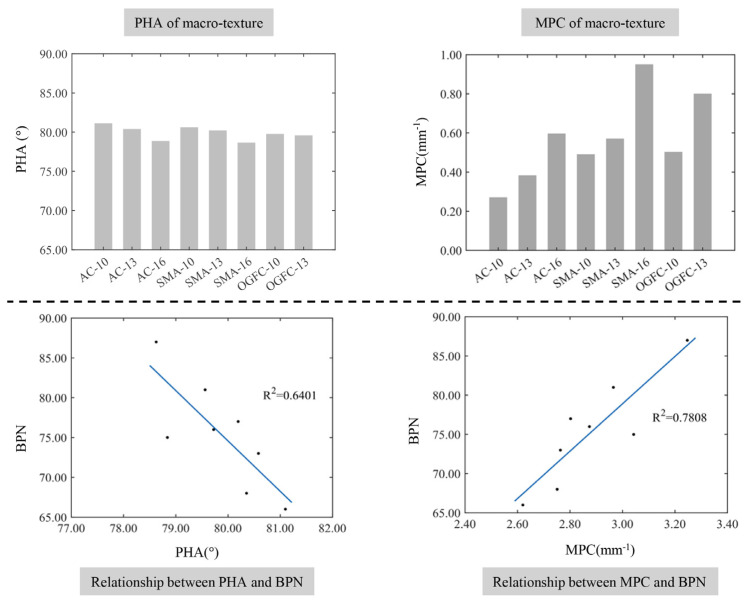
The relationship between the shape characteristics of the macro-texture protrusions and the BPN.

Figure 13 illustrates the height characteristic parameters of the macro-texture for different graded asphalt mixture specimens and their relationship with the BPN. It can be seen from Figure 14 that the MTD of the texture is consistent with the RMSD of the profile, and both show characteristics that are significantly related to the gradation type of the mixture and the maximum nominal particle size. The MTD and RMSD of the macroscopic texture increase with the increase in the maximum nominal particle size for mixtures with the same gradation type. The macro-texture height of the open-graded pavement is greater than that of the dense-graded pavement, which shows this order as OGFC > SMA > AC for the same maximum nominal particle size. At the same time, the BPN of asphalt pavement shows a significant positive correlation with the macro-texture height parameters. As the height of the macro-texture increases, the skid resistance of the pavement improves, which is due to the increased height of the macro-texture of asphalt pavement increasing the unevenness of the pavement surface and the energy loss increases using the elastic hysteresis in friction between the tire and the pavement. The fitting coefficient of the RMSD and the BPN is slightly larger than that of the MTD and the BPN, which indicates that *Rq* exhibits a more significant correlation with the BPN than the MTD. This can be attributed to the convex part of the pavement surface mainly affecting the elastic hysteresis and the RMSD amplifying the profile offset distance of the convex part.

#### 4.1.2. Protrusion Shape Feature Parameters

The relationships between the shape characteristic parameters of macro-texture protrusions and the BPN of different graded asphalt mixture specimens are demonstrated in Figure 14. The PHA and MPC also show a significant correlation with the mixture gradation and maximum nominal particle size. The profile peak of the pavement macroscopic texture is mainly caused by the irregular shape of the aggregate, and asphalt pavement tends to form a sharper convex peak as the proportion of the coarse aggregate or particle size of the aggregate is large, which leads to the larger MPC and smaller PHA. The increased amount of fine aggregate in the mixture leads to the larger PHA and a smaller MPC. This can be explained by the small and dense voids being easier to form on the surface of the pavement, and it is not easy to form a protruding profile peak. Interestingly, there is a negative correlation between the PHA and the BPN, and the fitting coefficient *R*^2^ is 0.6401. The MPC and BPN has a positive relationship, whose fitting coefficient *R*^2^ is 0.7808. On the one hand, the sharper macro-texture profile peak can lead to a greater contact stress and an increased elastic hysteresis generated during the contact between the rubber and the pavement surface. On the other hand, the increased sharpness of the macro-texture profile peak will cause a decrease in the contact area, which in turn reduces the frictional resistance caused by the adhesion.

#### 4.1.3. Comprehensive Distribution Characteristic Parameters

**Figure 15:**
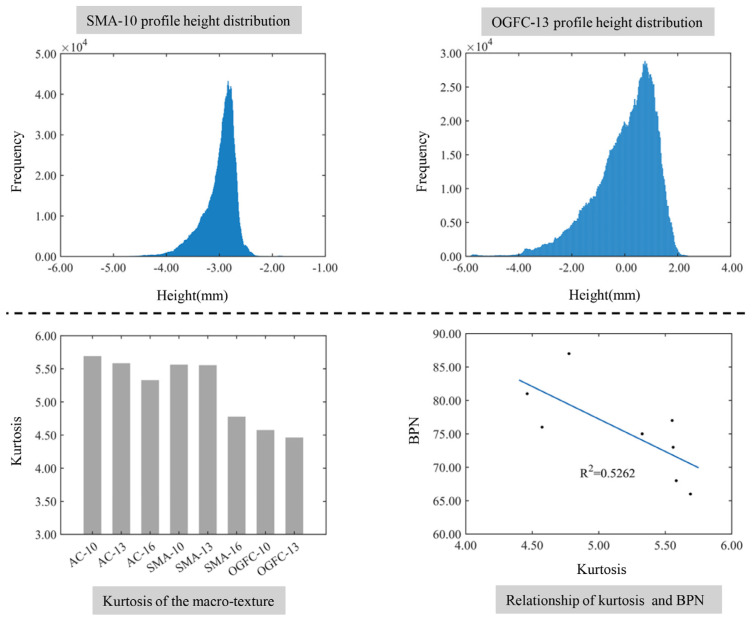
The relationship between the comprehensive distribution characteristics of the macro-texture and the BPN.

Figure 15 illustrates the relationship between the macro-texture comprehensive distribution characteristics and the BPN. It can be seen from Figure 15 that the elevation distribution of the surface profile for SMA-10 is more concentrated than that of OGFC-13, which is attributed to the fact that the kurtosis of the macro-texture for SMA-10 is greater than that of OGFC-13. The steeper elevation data of the pavement contour indicates that most of the contour elevations are distributed around mean values, and there are fewer elevation data deviated from the mean value. It can also be concluded that the kurtosis of the macroscopic texture is related to the gradation of mixture and the maximum nominal particle size. The kurtosis of the macro-texture for open-graded asphalt pavement is smaller than that of dense-graded asphalt pavement, which indicates that the distribution of data furthest from the mean is more dispersed in the elevation data of the open-graded pavement profile. This is consistent with the performance of larger void depths on open-graded pavement surfaces. The kurtosis of the macro-texture for asphalt pavement decreases slightly as the maximum nominal particle size increases, which can be explained by the increase in the amount of coarse aggregate leading to an increase in the amount of protruding aggregate on the pavement. 

Interestingly, the increased kurtosis of the macro-texture will lead to a decrease in the BPN, and the fitting coefficient is *R*^2^ = 0.5262. The degree of correlation is smaller than the height parameter of the macro-texture and the shape of the protrusion. This can be explained by the kurtosis of the macro-texture at the pavement surface being relatively large, and the distribution curve of the profile elevation being sharper than the normal distribution curve [41,42]. Therefore, the kurtosis of the macro-texture only shows a dispersion degree of the profile height distributed near the mean values and has no evident impact on the skid resistance of the pavement surface.

### 4.2. Microscopic Texture Features and Skid Resistance Analysis 

#### 4.2.1. Height Characteristic Parameters

**Figure 16:**
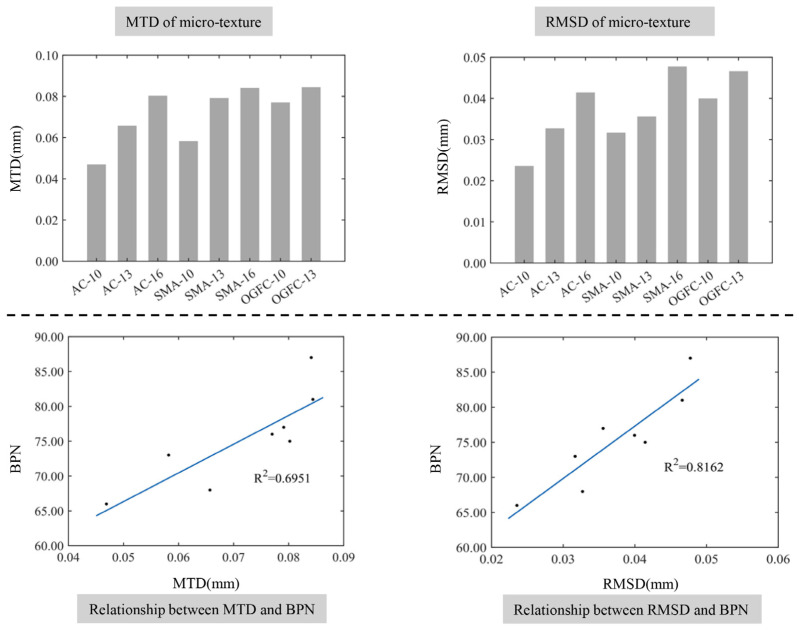
The relationship between the height characteristics of the micro-texture and the BPN.

**Figure 17:**
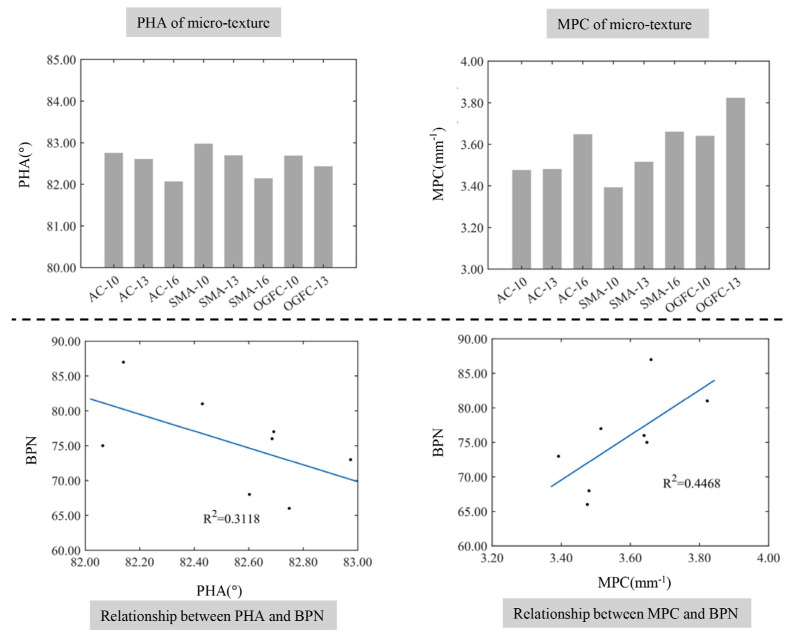
The relationship between the shape characteristics of the micro-texture protrusions and the BPN.

The microscopic texture of asphalt pavement mainly describes the microstructure of the aggregate surface, which is determined by the surface structure of the aggregate and the amount of asphalt. The relationship between the microscopic texture height characteristics and the BPN is demonstrated in Figure 16. It can be seen from Figure 17 that the height parameter of the microscopic texture is generally small, and increases with the addition of the maximum nominal particle size of the asphalt mixture. The height of the aggregate surface is related to the size of the aggregate, and the size of the surface structure of the aggregate with a larger particle size is larger. Therefore, the larger size and the amount of coarse aggregate in the asphalt mixture can lead to a larger height of the microscopic texture. 

In addition, there is a positive correlation between the characteristic parameters of the height of the micro-texture and the BPN. This is because the height of the micro-texture mainly affects the micro-cutting effect of pavement micro-bulges on the rubber. The deformation of the microscopic texture is large, and the microscopic texture is enveloped in the rubber as the tire is running on the road. Therefore, the increased height of the microscopic texture can increase the cutting depth of the rubber by the micro-bulge and the friction component caused by the cutting action of the micro-bulge, which can enhance the skid resistance of the pavement surface.

#### 4.2.2. Shape Feature Parameters of Textured Protrusions

**Figure 18:**
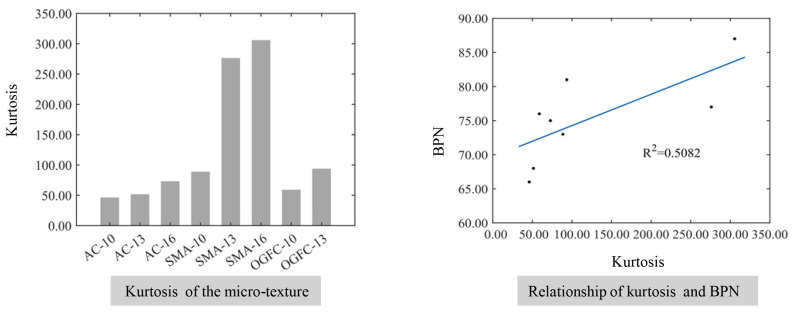
The relationship between the comprehensive distribution characteristics of the micro-texture and the BPN.

Figure 17 demonstrates the relationship between the shape characteristics of micro-textured protrusions and the BPN. The PHA of the microscopic texture for each specimen is relatively close to the MPC, the average PHA is 82.53°, and the difference between the maximum and minimum values is 0.81°. The mean value of MPC for the profile is 3.58 mm^−1^, and the difference between the maximum and minimum values is 0.43 mm^−1^. The PHA of the microscopic texture is related to the maximum nominal particle size within a small range of variation and decreases with the increase in the maximum nominal particle size. The addition of the maximum nominal particle size increases the MPC, and PHA on the surface of the SMA type mixture achieves the maximum, but the MPC is the smallest. This is due to the addition of lignocellulose into the SMA reducing the sharpness of the profile peak of its microstructure. In addition, according to Figure 18, the fitting coefficient *R*^2^ between the BPN and the shape parameter of the micro-texture is 0.3118 and the fitting coefficient *R*^2^ between the MPC and the BPN is 0.4468. The smaller fitting coefficient indicates that the sharpness of the profile peak of the micro-texture has little effect on the skid resistance of pavement.

#### 4.2.3. Comprehensive Distribution Characteristic Parameters

The relationship between the comprehensive distribution characteristics of the micro-texture and the BPN is shown in Figure 18. The kurtosis of the microscopic texture increases with the increase in the maximum nominal particle size, which is different from the kurtosis of the macroscopic texture. This can be explained by the microscopic texture of the pavement mainly describing the microstructure of the aggregate surface and the structure of the surface for large aggregates is relatively uniform and single compared with the variety of microstructures in the small size of aggregates. Therefore, the elevation distribution of microscopic texture is more concentrated with an increase in the maximum nominal particle size. In addition, the kurtosis of the micro-texture for the SMA-type mixtures is significantly greater than that of the AC and the OGFC due to the covering effect of fine aggregates and asphalt on the surface structure of coarse aggregates. At the same time, the fitting coefficient *R*^2^ between the kurtosis of the micro-texture and the BPN is 0.5082, which indicates that the kurtosis of the micro-texture has little effect on the skid resistance of the pavement surface.

### 4.3. Fractal Dimension of Asphalt Pavement and Skid Resistance Analysis

**Figure 19:**
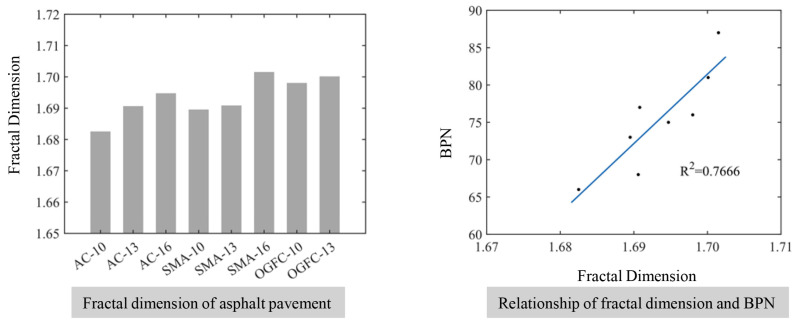
The relationship between the fractal dimension of the pavement surface and the BPN.

The fractal dimension of asphalt pavement reflects the irregularity of the pavement topography and self-similarity on a certain level [46]. Figure 19 illustrates the relationship between the fractal dimension of the pavement surface and the BPN. It can be seen that the fractal dimension of asphalt pavement is related to maximum nominal particle size and gradation type of the asphalt mixture, and the BPN increases as the fractal dimension increases, which indicates that an increased fractal dimension enhances the skid resistance. This can be attributed to the complexity of the pavement surface topography increasing and the texture topography becoming richer when the irregular bump distribution of the pavement increases. Interestingly, the roughness of the pavement increases with an increase in the macroscopic texture because microscopic texture of the pavement is not much different. When the maximum nominal particle size of the mixture increases or the gradation becomes sparser, the textures become coarser and the degree of self-similarity for surface topography increases, which leads to the increase in fractal dimension and an improvement of the skid resistance for asphalt pavement.

## 5. Conclusions and Recommendations

In this paper, three-dimensional laser image technology was used to extract and separate the high-precision feature information of macro- and micro-texture of asphalt pavement, and the Monte Carlo expectation method was used to calculate the MTD. On this basis, the relationship between pavement texture features and the skid resistance of asphalt pavement was also established through linear fitting. The conclusions can be drawn as follows.

(1) The median and parametric methods will identify part of the pavement texture profile as outliers, and the GESD method can more accurately identify outliers in the pavement texture.
(2) The signal after wavelet threshold denoising can retain most of the original signal features, but there are still some glitches in the denoised signal. The mean filter and median filter can both eliminate glitch points, and the median filter is in the middle of the wavelet threshold denoising and the mean filter methods, which is close to the original signal.
(3) The Monte Carlo expectation method can exactly calculate the MDT of reconstructed pavement, and the 98% and 2% parametric points of overall data as upper and lower limits of the Monte Carlo projection area is performed to obtain the MDT after the selection of the upper and lower limits.
(4) The height of the macro-texture is greater and sharper protruding peaks appear to enhance the skid resistance of the pavement as the maximum nominal particle size increases or the asphalt mixture changes from dense gradation to open gradation.
(5) The larger maximum nominal particle size of the asphalt mixture enriches the aggregate surface structure, reduces the amount of fine aggregate and asphalt, and increases the height and sharpness of the micro-texture of the pavement, which leads to an improved skid resistance of the pavement. The elevation distribution of the micro-texture has little effect on the skid resistance of the pavement.

This paper investigates the relationship between the characteristic of macro- and micro-texture and the skid resistance of asphalt pavement, which can present the interior mechanism and specific relationship between pavement textures and the skid resistance of asphalt pavement. Our future work will further consider more factors with a combination of the macro- and micro-texture to predict the skid resistance of asphalt pavement more comprehensively, use continuous friction measuring equipment (CFME) to determine the skid resistance of asphalt pavement as comparison, perform other materials of the rutting slab and statistical analysis including the response surface method (RSM) to validate the results of the method in this paper, and investigate the effect of aging and wear on roughness.

## Figures and Tables

**Table 1:** Basic physical properties of the origin bitumen.

Test Parameters	Value	Test Methods
Density, g/cm^3^	1.004	ASTM D70 [21]
Ductility at 25 °C, cm	88	ASTM D113-99 [22]
Viscosity at 135 °C, Pa·s	0.49	ASTM D4402 [23]
Soften point (R&B), °C	48.3	ASTM D36-95 [24]
Penetration @ 25 °C, 0.1 mm	56	ASTM D5-97 [25]
PG grade	64–22	ASTM D6373 [26]

**Table 2:** Gradation design of the three types of asphalt mixtures.

Sieve Hole Size/mm	AC			SMA			OGFC	
	16	13	10	16	13	10	13	10
16	91	100	100	91	100	100	100	100
13.2	78	95	100	67	99	100	99	100
9.5	63	77	95	47	72	99	78	99
4.75	38	53	60	21	32	56	28	68
2.36	24	37	44	16	25	31	21	21
1.18	16	27	32	15	23	25	17	17
0.6	11	19	23	13	19	21	14	14
0.3	9	14	16	11	15	17	11	11
0.15	6	10	11	10	14	15	7	7
0.075	5	6	6	8	12	12	5	5
Oil-stone ratio	4.4	4.7	4.9	5.4	6.0	6.2	4.8	4.9

## Data Availability

The data presented in this study are available on request from the corresponding author.

## References

[1] Wang Y., Yang Z., Liu Y., Sun L. (2019). The characterisation of three-dimensional texture morphology of pavement for describing pavement sliding resistance. Road Mater. Pavement Des..

[2] Guo W., Chu L., Yang L., Fwa T. (2023). Determination of tire rubber-pavement directional coefficient of friction based on contact mechanism considerations. Tribol. Int..

[3] Li J., Chen Z., Xiao F., Amirkhanian S.N. (2021). Surface activation of scrap tire crumb rubber to improve compatibility of rubberized asphalt. Resour. Conserv. Recycl..

[4] Rosenkranz A., Costa H.L., Baykara M.Z., Martini A. (2021). Synergetic effects of surface texturing and solid lubricants to tailor friction and wear—A review. Tribol. Int..

[5] Magnus C., Gulenc I.T., Rainforth W. (2022). Ambient dry sliding friction and wear behaviour of laser surface textured (lst) ti3sic2 max phase composite against hardened steel and alumina. Wear.

[6] Wang H.-P., Zhang M.-Q., Sun R.-X., Cui S.-J., Mo J.-L. (2023). Performance improvement strategy of the tbm disc cutter ring material and evaluation of impact-sliding friction and wear performance. Wear.

[7] Haritonovs V., Tihonovs J. (2014). Use of unconventional aggregates in hot mix asphalt concrete. Balt. J. Road Bridge Eng..

[8] Xu Y., Yang R., Chen P., Ge J., Liu J., Xie H. (2022). Experimental study on energy and failure characteristics of rubber-cement composite short-column under cyclic loading. Case Stud. Constr. Mater..

[9] Kogbara R.B., Masad E.A., Kassem E., Scarpas A.T., Anupam K. (2016). A state-of-the-art review of parameters influencing measurement and modeling of skid resistance of asphalt pavements. Constr. Build. Mater..

[10] Yang G., Wang K.C., Li J.Q. (2021). Multiresolution analysis of three-dimensional (3d) surface texture for asphalt pavement friction estimation. Int. J. Pavement Eng..

[11] Du Y., Weng Z., Li F., Ablat G., Wu D., Liu C. (2022). A novel approach for pavement texture characterisation using 2d-wavelet decomposition. Int. J. Pavement Eng..

[12] Jahanshahi M.R., Jazizadeh F., Masri S.F., Becerik-Gerber B. (2013). Unsupervised approach for autonomous pavement-defect detection and quantification using an inexpensive depth sensor. J. Comput. Civ. Eng..

[13] Puzzo L., Loprencipe G., Tozzo C., D’andrea A. (2017). Three-dimensional survey method of pavement texture using photographic equipment. Measurement.

[14] Moldovanu A., Huang L. (2018). Development of a portable circular texture meter for road texture depth measurement. J. Transp. Eng. Part B Pavements.

[15] Hoang N.-D., Nguyen Q.-L. (2019). A novel method for asphalt pavement crack classification based on image processing and machine learning. Eng. Comput..

[16] Alhasan A., Smadi O., Bou-Saab G., Hernandez N., Cochran E. (2018). Pavement friction modeling using texture measurements and pendulum skid tester. Transp. Res. Rec..

[17] Hofko B., Kugler H., Chankov G., Spielhofer R. (2019). A laboratory procedure for predicting skid and polishing resistance of road surfaces. Int. J. Pavement Eng..

[18] Li Q.J., Zhan Y., Yang G., Wang K.C. (2020). Pavement skid resistance as a function of pavement surface and aggregate texture properties. Int. J. Pavement Eng..

[19] Plati C., Pomoni M., Georgouli K. (2020). Quantification of skid resistance seasonal variation in asphalt pavements. J. Traffic Transp. Eng..

[20] Wang D., Zhang Z., Kollmann J., Oeser M. (2020). Development of aggregate micro-texture during polishing and correlation with skid resistance. Int. J. Pavement Eng..

[21] (2021). Standard Test Method for Density of Semi-Solid Asphalt Binder (Pycnometer Method).

[22] (2010). Standard Test Method for Ductility of Bituminous Materials.

[23] (2012). Standard Test Method for Viscosity Determination of Asphalt at Elevated Temperatures Using a Rotational Viscometer.

[24] (2017). Standard Test Method for Softening Point of Bitumen (Ring-and-Ball Apparatus).

[25] (2017). Standard Test Method for Penetration of Bituminous Materials.

[26] (2015). Standard Specification for Performance-Graded Asphalt Binder.

[27] Zhou B., Chu L., Yin C., Fwa T. (2022). Improved laboratory laser scanning setup and test procedure for 3-d pavement texture measurement. Measurement.

[28] Zou Y., Yang G., Cao M. (2022). Neural network-based prediction of sideway force coefficient for asphalt pavement using high-resolution 3d texture data. Int. J. Pavement Eng..

[29] Ding S., Yang E., Wang K.C., Wang G. Texture measurement based on 3d pavement surface images at sub-mm resolutioned. Proceedings of the International Conference on Transportation and Development: Airfield and Highway Pavements.

[30] Katicha S.W., Flintsch G., Bryce J., Ferne B. (2014). Wavelet denoising of tsd deflection slope measurements for improved pavement structural evaluation. Comput.-Aided Civ. Infrastruct. Eng..

[31] Atef A.H., Rashed M.A. (2023). Gpr ringing suppression using lateral outliers’ swap filter. J. Appl. Geophys..

[32] Bashar M.Z., Torres-Machi C. (2022). Deep learning for estimating pavement roughness using synthetic aperture radar data. Autom. Constr..

[33] Yuwono B. (2015). Image smoothing menggunakan mean filtering, median filtering, modus filtering dan gaussian filtering. Telemat. J. Inform. Dan Teknol. Inf..

[34] Katicha S.W., Mogrovejo D.E., Flintsch G.W., De León Izeppi E.D. (2015). Adaptive spike removal method for high-speed pavement macrotexture measurements by controlling the false discovery rate. Transp. Res. Rec..

[35] Deng Q., Zhan Y., Liu C., Qiu Y., Zhang A. (2021). Multiscale power spectrum analysis of 3d surface texture for prediction of asphalt pavement friction. Constr. Build. Mater..

[36] Serigos P.A., De Fortier Smit A., Prozzi J.A. (2014). Incorporating surface microtexture in the prediction of skid resistance of flexible pavements. Transp. Res. Rec..

[37] Chen S., Liu X., Luo H., Yu J., Chen F., Zhang Y., Ma T., Huang X. (2022). A state-of-the-art review of asphalt pavement surface texture and its measurement techniques. J. Road Eng..

[38] Kheradmandi N., Mehranfar V. (2022). A critical review and comparative study on image segmentation-based techniques for pavement crack detection. Constr. Build. Mater..

[39] Barnett R., Reynolds P., Lester W. (1991). Monte carlo algorithms for expectation values of coordinate operators. J. Comput. Phys..

[40] Reiter D. (2008). The monte carlo method, an introduction. Comput. Many-Part. Phys..

[41] China S., James D.E. (2012). Comparison of laser-based and sand patch measurements of pavement surface macrotexture. J. Transp. Eng..

[42] Dan H.-C., Bai G.-W., Zhu Z.-H., Liu X., Cao W. (2022). An improved computation method for asphalt pavement texture depth based on multiocular vision 3d reconstruction technology. Constr. Build. Mater..

[43] Zhihong M., Guo C., Yanzhao M., Lee K. (2011). Curvature estimation for meshes based on vertex normal triangles. Comput.-Aided Des..

[44] Chen L., He Z.-Y., Chen H.-B. (2016). Road performance research on cold recycled pavement base with foamed asphalt on the basis of fractal dimension. J. Highw. Transp. Res. Dev..

[45] Moghaddam R.F., Cheriet M. (2012). Adotsu: An adaptive and parameterless generalization of otsu’s method for document image binarization. Pattern Recognit..

[46] Liu C., Zhan Y., Deng Q., Qiu Y., Zhang A. (2021). An improved differential box counting method to measure fractal dimensions for pavement surface skid resistance evaluation. Measurement.

